# Immunization with SP_1992 (DiiA) Protein of *Streptococcus pneumoniae* Reduces Nasopharyngeal Colonization and Protects against Invasive Disease in Mice

**DOI:** 10.3390/vaccines9030187

**Published:** 2021-02-24

**Authors:** Antonio J. Martín-Galiano, María S. Escolano-Martínez, Bruno Corsini, Adela G. de la Campa, José Yuste

**Affiliations:** 1 Centro Nacional de Microbiología, Instituto de Salud Carlos III (ISCIII), 28220 Madrid, Spain; memartinez@alumni.unav.es (M.S.E.-M.); bcorsini@ucm.es (B.C.); agcampa@isciii.es (A.G.d.l.C.); 2 Presidencia Consejo Superior de Investigaciones Científicas, 28006 Madrid, Spain; 3 CIBER de Enfermedades Respiratorias (CIBERES), 28029 Madrid, Spain

**Keywords:** *Streptococcus pneumoniae*, reverse vaccinology, hypothetical protein, DiiA, sepsis, carrier state, protein vaccine, SPD_1789, Spr1806

## Abstract

Knowledge-based vaccinology can reveal uncharacterized antigen candidates for a new generation of protein-based anti-pneumococcal vaccines. DiiA, encoded by the sp_1992 locus, is a surface protein containing either one or two repeats of a 37mer N-terminal motif that exhibits low interstrain variability. DiiA belongs to the core proteome, contains several conserved B-cell epitopes, and is associated with colonization and pathogenesis. Immunization with DiiA protein via the intraperitoneal route induced a strong IgG response, including different IgG subtypes. Vaccination with DiiA increased bacterial clearance and induced protection against sepsis, conferring 70% increased survival at 48 h post-infection when compared to the adjuvant control. The immunogenic response and survival rates in mice immunized with a truncated DiiA version lacking 119 N-terminal residues were remarkably lower, confirming the relevance of the repeat zone in the immunoprotection by DiiA. Intranasal immunization of mice with the entire recombinant protein elicited mucosal IgG and IgA responses that reduced bacterial colonization of the nasopharynx, confirming that this protein might be a vaccine candidate for reducing the carrier rate. DiiA constitutes an example of how functionally unannotated proteins may still represent promising candidates that can be used in prophylactic strategies against the pneumococcal carrier state and invasive disease.

## 1. Introduction

*Streptococcus pneumoniae* is one of the most prevalent etiological agents of both invasive and noninvasive disease including pneumonia, meningitis and sepsis. This relevant microorganism remains one of the most deadly and costly pathogens with a rate over one million deaths per year worldwide affecting mainly children under five years old and elderly adults [1,2]. Emergence of multidrug resistant clinical isolates and the high morbidity and mortality rates have driven attention to vaccine development. The current vaccine formulations are based on selected capsular types that may be conjugated to a carrier protein in order to elicit protection against the pediatric and adult population [3]. However, these vaccines have several limitations. Up to 100 different capsular polysaccharides have been described [4]; therefore, serotypes included in a single formulation only represent a small fraction of the total that exist. Serotype replacement linked to the long-term pressure exerted by vaccination has led to emerging infective genotypes that have previously incorporated alternative capsules, which eventually tend to dominate the clinical cases [5,6,7]. An additional limitation is the high cost of conjugated vaccine preparations including numerous capsular serotypes that may be unaffordable for low-income families or countries. Overall, capsule-based protective strategies against pneumococcus are leading to a “red-queen” scenario, in which the bacteria evolves to circumvent the vaccine protective effect as new formulations are developed in a never-ending fashion [8].

An alternative prophylactic strategy consists of vaccinating with universal epitopes carried by single proteins, peptides or cocktails of them. These proteins must ideally show the following features: (1) exposure to the outside; (2) ability to produce a strong and complete immunogenic response in the host; (3) limited allelic variability; (4) presence in the highest possible number of clinical isolates; (5) playing a role in virulence in one or more stages of infection [9]. Pneumococcal proteins classically investigated as antigens were further supported by the use of the ANTIGENome technique [10], which reveals the proteome subset recognized by antisera of patients and carriers. However, the most promising antigens, according to ANTIGENomic results, show extreme allelic variability that hinders their applicability as antigens. For instance, pneumococcal surface protein A (PspA) variants are clustered into two clades and six families with high sequence dispersion [11]. Choline binding protein A (CbpA) shows up to 11 mosaic domain combinations [12]. Pilus proteins, among others, are immunogenic but present in only a fraction of clinical isolates [13]. Pneumococcal histidine triad protein A (PhtA) is truncated in some strains [14]. This variability is a consequence of the balance between the confronting forces of function maintaining and immune system evasion, which eventually shape outer antigenic proteins relevant in virulence.

Reverse vaccinology implies the utilization of immunoinformatic techniques in association with omic data to rationally find antigens in primary human pathogens [15,16,17,18]. The genome of several *S. pneumoniae* strains has been fully sequenced since 2001 [19,20], and a strong body of knowledge about its surface virulence factors is available [21,22]. Hypothetical proteins represent a sizable fraction of the proteome in all bacterial species that are systematically flagged by design in target selection protocols despite the fact that some of them are part of the core proteome. In this sense, several fundamental aspects of the dimorphic invasion-involved protein A (DiiA) remain unresolved; therefore, this protein could still be classified as a “hypothetical protein”. DiiA is a pneumococcal surface protein encoded by the sp_1992 locus in the TIGR4 strain that albeit may potentially become a vaccine candidate. This protein shows two major allelic variants containing either one (R2) or two (R1 and R2) aminoterminal repeats [23]. DiiA is essential for optimal colonization and sepsis in animal models. Clinical isolates carrying the long allele are epidemiologically associated (1.8-fold higher) with invasive pneumococcal disease, one of the most life-threatening pathologies of this pathogen, but not to noninvasive diseases [23]. The nonrepeat (NR) region located between the N-terminal repeat zone and the C-terminal LPxTG cell wall anchor is able to bind in vitro to collagen and lactoferrin, two important proteins of the innate immunity. The sp_1992 gene is upregulated in response to high manganese concentration [24] and in the pleural environment [25].

In this work, we demonstrate that DiiA is able to induce an immunoprotective response reducing the carrier state and protecting against pneumococcal sepsis, suggesting that this protein can be deemed a promising antigenic vaccine candidate.

## 2. Materials and Methods 

### 2.1. Epitope/Immunogenicity Prediction

A battery of protocols was applied on the 330-residue TIGR4 DiiA protein using default thresholds. Contiguous B-cell epitopes were predicted by AAPPred-SVM1 and SVM2 [26] with a score ≥0, ABCPred [27] with a score ≥0.8, BepiPred [28] with a cutoff of ≥0.35, BepiPred-2.0 [28] with a cutoff of ≥0.5, Kolaskar’s antigenicity [29] with a score threshold of ≥0.988, LBEEP [30] using a window length of 15 residues and a score of 0.7, and SVMtrip [31], applying a score of ≥0.35.

T-cell epitopes of human leukocyte antigen (HLA) class II were predicted by netMHCIIpan 4.0 [32] on five supertype alleles [33] applying an IC50 ≤ 500 nM as threshold [31]. Protein toxicity was predicted by ToxDL [34]. Potential allergenicity was predicted by AllerTOP 2.0 [35] and by AlgPred [36] by mapping IgE epitopes, MEME motifs and BLAST search to allergen representative peptides.

### 2.2. Immunization Experiments in Mice for Antibody Response Determination

BALB/c mice were bred by our institutional (ISCIII) animal facility. All mice used were 8-16 weeks old and, within each experiment, groups of mice were matched for age and sex. Animal experiments were performed at ISCII in accordance with Spanish legislation (RD 53/2013) and EU regulations (218/63/EU). The Animal Care and Use Committee of ISCIII (PROEX 218/15) approved animal experiments performed in this work.

The pneumococcal DiiA−R1R2 or DiiA−NR proteins used for immunization studies were obtained and purified as previously described [23]. Briefly, PCR gene fragments were cloned into pET28 plasmid and transferred to expressing BL21-CodonPlus cells (Agilent Technologies, Inc, Santa Clara, CA, USA). After protein expression, cells were lysed and ultracentrifuged. The resulting supernatant was applied to a HisTrap HP column (GE Healthcare, Madrid, Spain) for affinity chromatography and then to a HiTrap HP column (GE Healthcare) for anion exchange chromatography. The protein solution was desalted and eluted with 100 mM NaCl, 1 mM EDTA, 20 mM Tris-ClH (pH 7.5). Proteins were prepared for animal inoculations at 20 μg in Alum (Alhydrogel; aluminum hydroxide, InvivoGen®, Toulouse, France) as the adjuvant in a 1:1 proportion. For systemic determination of IgG response, groups of 5 mice were immunized by intraperitoneal (IP) inoculations of 200 μL of Alum alone or 200 μL of each protein preparation in Alum adjuvant on days 0, 7 and 14 as previously described [37,38]. Animals were euthanized on day 21 and blood was collected from cardiac puncture and pooled sera conserved at −80 °C for further in vitro assays. For mucosal determination of IgA and IgG responses, groups of 5 mice were immunized by the intranasal (IN) route with 20 μL of Alum alone or 20 μL of each protein mixed with Alum on days 0, 7 and 14 as described above. Animals were euthanized on day 21 and nasopharyngeal lavage fluid of each mouse was collected as previously described [39] and conserved as pooled at −80 °C for further in vitro assays.

### 2.3. Enzyme Linked Immunosorbent Assays

Enzyme linked immunosorbent assays (ELISA) were used to detect Ig subclasses as previously published [38,40]. Briefly, specific antibody titers in pooled sera from five mice of each group were measured using 96-well polystyrene Maxisorp plates (Nunc) coated with 0.5 μg of either purified DiiA−R1R2 or DiiA−NR proteins for 2 h at 37 °C and blocked with PBS-2% bovine serum albumin solution as previously described [38,40]. Different dilutions of pooled sera from mice immunized with only Alum as adjuvant, or DiiA protein variants mixed with Alum, were performed. The volume of 50 µL of sera was added to the correspondent wells. Bound antibodies were detected by using horseradish peroxidase (HRP)-conjugated goat anti-mouse IgG, IgG1, IgG2a, IgG2b, IgG3 and IgA (Santa Cruz Biotechnology, Inc, Dallas, TX, USA) for 30 min and developed using o-phenylenediamine (Sigma-Aldrich, Madrid, Spain) before determining the OD_492_ using a microtiter plate reader (Anthos 2020, Biochrom, Cambridge, UK).

### 2.4. Western Blot

Different amounts of purified DiiA−R1R2 protein were loaded into a 4–20% gradient acrylamide gel, run, and transferred to a polyvinylidene fluoride membrane using a Trans-Blot Turbo Transfer System (Bio-Rad, Madrid, Spain). The membrane was blocked with Superblock buffer (Thermo Fisher Scientific, Madrid, Spain), hybridized at room temperature for two hours with a 1:500 dilution of pooled antiserum, washed and hybridized by 2 h with peroxidase conjugated-protein G (Thermo Fisher Scientific). Bands were revealed after incubation by 5 min with SuperSignal West Pico Chemiluminiscent Substrate (Thermo Fisher Scientific).

### 2.5. Bacterial Strains and Growth Conditions

The *S. pneumoniae* isolate used in this study was strain TIGR4 [serotype 4; amoxicillin MIC = 0.015 µg mL^−1^; erythromycin MIC = 0.03 µg mL^−1^, levofloxacin MIC = 0.5 µg mL^−1^, tetracycline MIC = 0.12 µg mL^−1^, chloramphenicol MIC = 1 µg mL^−1^]. The pneumococcal strain was cultured at 37 °C in 5% CO2 on blood agar plates or in Todd-Hewitt broth supplemented with 0.5% yeast extract to an optical density at 580 nm (OD_580_) of 0.4–0.5 (approximately 10^8^ colony-forming units [CFU]/mL) and stored at −80 °C in 10% glycerol as single-use aliquots.

### 2.6. Vaccination Experiments in Mice for Protection and Bacterial Clearance

Protection experiments against sepsis were performed in groups of 10 mice immunized as previously described, followed by IP challenge on day 21 with 10^5^ CFU/per mouse of strain TIGR4. Bacterial counts were determined during the first 24 h and 48 h from blood samples (6 μL per mouse) obtained from the tail vein of infected animals as previously described [38,41]. The development of disease was monitored daily during 1 week, and mice were sacrificed when they exhibited severe signs of disease. To test the effect of vaccination with DiiA−R1R2 in nasopharyngeal colonization, immunized mice by the IN route were also intranasally infected on day 21 with 10 μL containing 10^6^ CFU of TIGR4 strain. After five days, mice were euthanized and nasopharyngeal lavage fluid of each mouse was obtained and different dilutions were cultured on blood agar plates for bacterial counts determination.

## 3. Results

### 3.1. DiiA Is a Potential Vaccine Candidate

Among the principal pneumococcal surface proteins detected by ANTIGENome, SP_0107 and SP_1992 (DiiA) are the only ones that do not show any known InterPro domain in the section exposed to the medium. Thus, they are potential antigens to control pneumococcal diseases that have not been analyzed so far.

**Figure 1:**
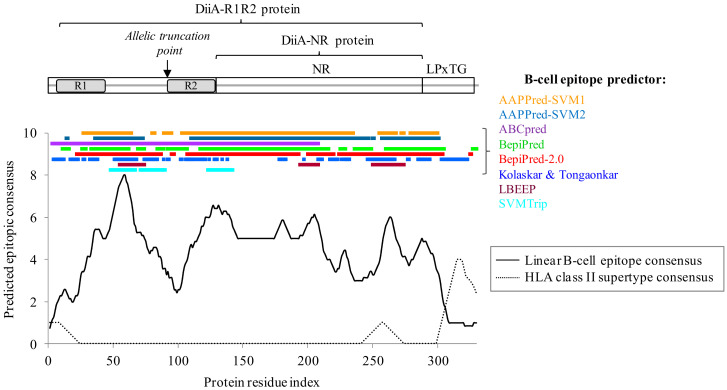
DiiA domain organization and B-cell/HLA class II epitope prediction. The predicted epitope consensus is the resultant of applying a window of seven residues for B-cell epitopes and 15 residues for HLA class II epitopes, their respective average lengths.

We took advantage of previous knowledge of DiiA and several additional lines of evidence that reinforce the notion that DiiA is a promising antigen vaccine candidate. The protein is encoded by a gene from the pneumococcal core genome and shows limited allelic variability, in particular when compared to other surface proteins such as CbpA and PspA as deduced by genomic and PCR data. The *diiA* gene was present in the fully complete genomes of 80 *S. pneumoniae* isolates (55 unique STs) available in the Assembly database (Last Accession: 30th December 2020). In this genomic subset, the predicted protein homolog sequences were conserved at an average ~98% identity with respect to SP_1992 in both long (containing both R1 and R2 repeats, 58% isolates) and short (containing only the R2 repeat, 38% isolates) alleles. Strikingly, in two isolates, a premature stop codon rendered a truncated protein of 151 residues, spanning just the repeat zone. This is probably a protein variant released into the medium, in contrast to the canonical cell wall-anchored one, a dual nature that has also been reported for some CbpG homologs [42]. Comparable results were obtained when the study was expanded to the 8315 *S. pneumoniae* isolates (1058 unique registered STs) in the RefSeq database (https://www.ncbi.nlm.nih.gov/RefSeq), in which the gene was detected (identity ≥75%, coverage ≥70%) in 88.1% genomes. This prevalence rate is still an underestimation because a small genome subfraction still carries the small truncated ~151 residue gene version. Moreover, some homologs may be artifactually lost because most of these genomes are in a “contig” status. Allele variability is also related to disease because clinical isolates carrying the long full version were significantly associated to invasive pneumococcal disease (IPD) in humans [23]. DiiA is involved in systemic infection as we have recently demonstrated using different animal models of disease including defective strains lacking different fragments of the protein [23]. Moreover, immunogenicity was also supported by sequence-based prediction using a consensus of eight epitope and immunogenicity predictors. The protein showed a highly global B-cell antigenic character because 70.9% of the sequence extension was predicted to convey B-cell epitopes according to ≥50% of the algorithms utilized (Figure 1). Notably, the strongest B-cell epitopic peak located at the inter-repeat zone with the agreement of 7–8 methods for the 12mer 51-KSTVKAPAQRVD-62 that was fully conserved in all isolates with complete genomes carrying this region, but one case that showed the S52P change. In any case, antigenicity was a sequence-distributed quality of the DiiA protein. Other candidate B-cell epitopic zones (123–142, 178–183, 200–208 and 260–267 spans, predicted by ≥6 methods) are present and also conserved in most isolates.

Mature antisera mostly require the activity of T helper lymphocytes activated through epitopes bound to human leukocyte antigen (HLA) class II molecules by professional antigen presenting cells. However, the protein was essentially devoid of epitopes binding with high-intermediate affinity to HLA class II allelic supertypes, which cover 90% human population. The only exception was the C-terminal cell-wall anchor motif that, however, should be avoided in the final antigen product due to potential problems concerning solubility and cross-reactivity to microbiota.

Concerning safety, the protein was predicted as nontoxic and nonallergenic because it did not contain any experimentally proven IgE epitope nor shared similarity to any known allergen.

Altogether, the DiiA protein satisfied most standard reverse vaccinology criteria as an anti-pneumococcal humoral antigen, which warrants experimental verification.

### 3.2. DiiA Protein Elicits a Strong and Diverse Immunogenic Response after Parenteral Immunization

**Figure 2:**
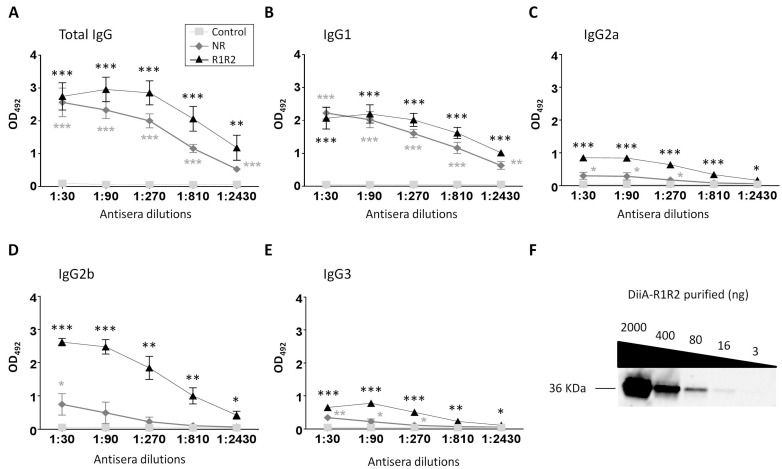
Antibody levels after immunization with control, DiiA-NR or DiiA-R1R2 preparations. Total IgG (**A**), IgG1 (**B**), IgG2a (**C**), IgG2b (**D**) and IgG3 (**E**). Pooled antiserum extracted from surviving immunized mice was used. Data are expressed as the average and standard deviation of three samples. Statistical significance of absorbance measures was analyzed respect to controls by Student’s t-test. * *p* < 0.05; ** *p* < 0.01; *** *p* < 0.001. (**F**) Verification of antiserum specificity by Western blot. Five-fold serial dilutions of purified protein and 1:500 serum dilution were utilized.

Based on promising computational results, the immunogenic capacity of DiiA was investigated experimentally. For that, two recombinant protein variants constructed on the TIGR4 background strain were used: DiiA−R1R2 (residues 1–287) containing both repeats and DiiA−NR, in which the region containing the repeats (residues 11–129) was deleted. The later lacks the B-cell epitope exclusive of the N-terminal zone, whereas it still harbors the remaining epitopes. The C-terminal cell wall anchor was deleted in both recombinant proteins to avoid insolubility and potential cross-reaction. In the two cases, proteins were independently mixed with Alum as co-adjuvant prior to injection. After immunization with three doses of 20 µg of protein each and spaced by one week, the two DiiA variants were more immunogenic than the negative control (Alum alone), whose antisera recognized purified DiiA−R1R2 at an almost negligible amount. As supported by the exclusive presence of a predicted immunodominant B-cell epitope, immunization with DiiA−R1R2 produced a stronger and more diverse response in terms of IgG type levels than DiiA−NR (Figure 2A–E). In particular, the induced IgG2a and IgG2b response associated with protection against carbohydrate antigens was noticeably higher for DiiA−R1R2 than the ones induced by DiiA-NR. Antisera were able to recognize purified DiiA−R1R2 by Western blot in the low nanogram amount range, indicative of a sensitive response (Figure 2F and Figure S1).

### 3.3. DiiA−R1R2 Protects Against Sepsis

**Figure 3:**
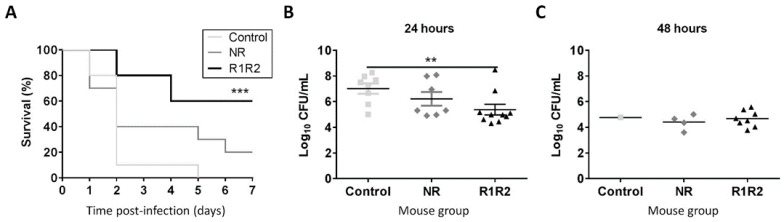
Protection against pneumococcal sepsis. (**A**) Time-course survival rates for sepsis in immunized mice after bacterial inoculation. CFU/mL counts in blood at 24h (**B**) and 48h (**C**) post-infection via intraperitoneal are shown. Statistical significance of survival was analyzed by the log rank (Mantel-Cox) test whereas CFU/mL was analyzed by Student’s t-test. ** *p* < 0.01; *** *p* < 0.001.

The protective capacity of the immunogenic response induced by the DiiA-based formulations was tested in a murine sepsis model of infection. Pre-immunized mice were challenged with direct intraperitoneal injection of TIGR4 bacteria. After 48h infection, 80% of DiiA−R1R2-immunized mice were alive, in contrast to 10% in control mice. Only 40% mice vaccinated with DiiA−NR version survived at this stage, which was not statistically significant (*p* = 0.22). The protective reduction observed with DiiA−NR respect to DiiA−R1R2 mirrored the lower immunogenic response that it elicited (Figure 2). Protection was observed even at 168 h (seven days); the fraction of surviving immunized mice was 60% for DiiA−R1R2 and 20% for DiiA−NR proteins, whereas all control mice succumbed to the TIGR4 sepsis infection. Thus, a 60–70% increment in survival rate in sepsis was achieved by utilizing DiiA−R1R2 (Figure 3A).

When the bacterial proliferation in blood at 24 h post-infection was assessed in vaccinated mice, CFU/mL counts were 1.6-log lower in mice immunized with DiiA−R1R2 than in the control group (5.4 ± 1.3 vs. 7.0 ± 1.2 logs, respectively; average ± standard deviation) (Figure 3B). At 48 h, when the majority of mice within the lethal control group were already dead, bacterial counts in DiiA−R1R2 mice were stabilized below 10^5^ CFU/mL in the blood of the surviving 80% DiiA−R1R2 mice, as happens with the 40% DiiA−NR mice and the only control mice able to reach this point (Figure 3C). The fact that the protective effect was similar in most of mice of the same group suggests that the high immunogenic response levels to DiiA as antigen, observed in the pool sera by ELISA, was very likely comparable within animals. These results confirmed that vaccinated mice were able to counteract the pneumococcal infection and justify why most of these mice survived in the following days.

### 3.4. DiiA−R1R2 Reduces Colonization in the Upper Respiratory Tract

**Figure 4:**
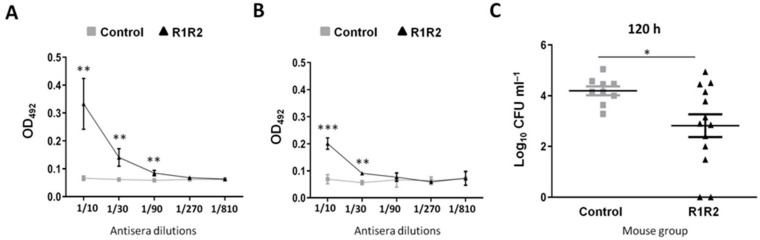
Protection against intranasal colonization. Antibody levels of IgA (**A**) and total IgG (**B**) after immunization with control, or DiiA−R1R2 preparations. Data are expressed as the average and standard deviation of triplicate samples. Bacterial load in nasopharyngeal lavage at 120 h post-colonization in groups of at least nine mice. (**C**). Statistical significance was analyzed by Student’s t-test. * *p* < 0.05; ** *p* < 0.01; *** *p* < 0.001.

Another serious clinical concern is the high rate of carrier individuals in the community, a prerequisite for transferring the infection to the at-risk population. Thus, based on the remarkably higher immunoprotective activity of DiiA−R1R2 with respect to DiiA−NR, we selected the former protein variant to explore its capacity to decrease nasopharyngeal colonization. First, the humoral immunogenicity of DiiA−R1R2 inoculated through the intranasal route was quantified. The nasopharyngeal wash of three-dose immunized mice contained not only higher concentrations of IgA (Figure 4A)—the main immunoglobulin in mucosal membranes—but also of IgG (Figure 4B) when compared to control mice. To check whether this immunogenic response actually contributes to preventing colonization in vivo, TIGR4 was inoculated intranasally. The bacterial load after 120 h (five days) of intranasal inoculation decreased one order of magnitude (4.0 ± 0.7 vs. 3.0 ± 1.3 logs, respectively) in immunized mice with respect to the control group (*p* = 0.04, Student’s t-test) (Figure 4C). These results indicate that immunization with DiiA−R1R2 reduces pneumococcal colonization in the nasopharyngeal tract.

## 4. Discussion

Prevention of invasive pneumococcal disease linked to high carrier rates of *S. pneumoniae* by vaccination is a major topic in medical microbiology. One of the major disadvantages of current used pneumococcal vaccines, based in polysaccharides, is capsular switching. This is a phenomenon that appeared promptly after the introduction of pneumococcal conjugate vaccines resulting in a serious concern worldwide. These strains may become established as frequent serotypes by avoiding vaccine-induced immunity due to acquisition of capsular genes from nonvaccine serotypes [5,43,44]. To circumvent these problems, the majority of efforts that are being made in the field focus on the discovery and characterization of conserved protein-based antigen candidates that may protect against *S. pneumoniae*.

However, further limitations associated with the use of classical immunogenic surface proteins of *S. pneumoniae* have increased interest in antigens ignored so far and found after deep exploration of postgenomic data. In this study, the DiiA protein has been feature-selected for analysis and proven as an immunoprotective antigen against nasopharyngeal colonization and systemic disease. The immunogenicity of DiiA in carriers and patients was previously revealed by ANTIGENome analysis and studied in this work by a consensus of bioinformatic tools. In particular, the prioritization of DiiA was justified by the presence of a cell wall anchor domain, pneumococcus-exclusivity, belonging to the core proteome of the species, low allelic variability, related epidemiologically to invasive disease, binding to important host proteins and its role in pathogenicity demonstrated in animal models [23]. Furthermore, the sp_1992 locus is up-regulated in response to manganese [24] which is important in providing defense against oxidative stresses in *S. pneumoniae* [45]. Furthermore, this gene is regularly present in pathogenic *S. pneumoniae* in contrast to related commensal species [46]. Due to the wide conservation of the gene encoding DiiA, which is present in virtually all *S. pneumoniae* isolates, it has been suggested as a target gene for identification in *S. pneumoniae* clinical isolates [47]. DiiA may therefore have prophylactic and diagnostic applications despite the fact that some of its functional aspects, notably the natural ligand of the repeats, still remain unknown.

DiiA immunogenicity was experimentally confirmed in a modular-dependent manner using animal models and quantified in a detailed way. Immunization experiments were performed using Alum because this adjuvant has been approved for human use and is widely utilized in studies evaluating pneumococcal proteins, although alternative adjuvants may be tested in future experiments [48,49,50]. DiiA promotes a complete immunogenic response involving the production of several IgG subtypes. These results are of great interest because current polysaccharide-based vaccines elicit some of these IgGs subtypes. In this sense, the 23-valent polysaccharide vaccine is predominantly of the IgG_2_ subclass [51,52], whereas pneumococcal conjugate vaccines induce higher amounts of IgG2 in adults and IgG1 in children [51,53,54]. Immunoinformatic and animal model results indicate that the repeat zone is important for protection. For example, the most frequently identified B-cell epitope according to eight predictors locates at this region, and the response induced by the full DiiA response exclusively involves different IgG2 subtypes, related to carbohydrate recognition. The later finding suggests that the N-terminal region exerts an additional immunomodulatory effect. This may be due to repeats that are potentially able to recognize carbohydrate-containing patterns.

DiiA conferred protection to mice against invasive pneumococcal challenge. The bacterial load in the bloodstream of DiiA-immunized mice significantly diminished compared to control mice immunized only with Alum, which finally succumbed to the infection. Protection was significantly weaker when immunization was performed with the DiiA−NR section. These results are in agreement with allelic epidemiological data associated with the presumed role in pathogenesis of this protein [23]. Hence, the engineered pneumococcal strain lacking only the repeats experienced a modest reduction in virulence in a sepsis model, whereas the full *ΔdiiA* mutant was essentially unable to persist in blood [23]. Vaccine antigens based on bacterial proteins, however, show difficulty in providing a clear impact on carriage in vaccinated children, as observed for the serogroup B meningococcal vaccine [55,56]. Our results demonstrate that the DiiA protein may contribute to decrease the pneumococcal carrier state and to prevent invasive disease in at risk populations. These results are also supported by a reduction of bacterial load in the nasopharynx, which might be of importance for preventing transmission from children to the elderly population in cases of broad implementation. This is important from the virulence perspective because nasopharyngeal colonization is the first step of the pathogenesis process and is a prerequisite for developing invasive pneumococcal disease [57].

The most immunogenic proteins of *S. pneumoniae*, such as CbpA and PspA, elicit strong responses but show radical interstrain variability [11,12] that hamper its utility as universal vaccine antigens. Such CbpA and PspA variability makes also difficult a direct automated comparison between the subfamilies of these proteins and DiiA alleles. This problem might be solved by combining in a single vaccine formulation, highly immunogenic multi-allele proteins with other proteins such as DiiA that show less interstrain variability. An antigenic cocktail containing several protein candidates of different natures would ameliorate their respective antigenic weaknesses among them, including limited sequence conservation and conditional protein expression during critical infection stages. Based on these limitations, the combination of pneumococcal proteins, instead of single proteins, seems to be the best approach for future pneumococcal vaccines as has been demonstrated to fight serogroup B meningococcal strains [58].

We have observed that some bona fide SP_1992 homologs are annotated as pseudogenes in protein databases. Nevertheless, several theoretical and experimental evidences contradict this statement. DiiA follows the classical “repeat–unstructured stalk–anchor” architecture typical of surface adhesins. An intact copy of the gene, showing low mutational rate, is present in a vast majority of pneumococcal isolates. Epidemiological and biochemical traits have been associated with protein variants. The gene is transcriptionally active under relevant environmental conditions. Moreover, homolog repeats were detected through a hidden Markov model in surface proteins of many bacterial species related to commensalism and invasive disease [23,59], suggesting that DiiA is a remote member of a disperse superfamily of exposed proteins in gram-positive bacteria.

*S. pneumoniae* appears to undergo strong selective pressure to maintain the *diiA* gene and sequence, which would decrease the chances of vaccine escape. DiiA is exclusive of this microorganism, absent even in the closest species *Streptococcus pseudopneumoniae*, indicating acquisition of the *diiA* gene occurred at the dawn of the *S. pneumoniae* speciation. DiiA therefore embodies the ideal of a prophylactic “magic bullet” directed against pneumococcus with expected negligible effects on commensal streptococci.

In conclusion, the utilization of reverse vaccinology can thus open the door to the identification of promising candidates annotated with vague descriptors at most, such as “uncharacterized surface protein”. Because these new players would enrich the activity of vaccine cocktails, we propose the inclusion of DiiA, or some of its components, in future protein-based formulations against pneumococcal carriage and invasive disease.

## 5. Conclusions

Alternative protein-based vaccine formulations to guard against pneumococcal disease have been reported in the last decade. However, hypothetical proteins have been disregarded mainly due to the lack of fundamental information. In this respect, DiiA is a recently characterized important virulence factor of *S. pneumoniae* playing a role in the establishment of colonization and sepsis in animal models. Numerous omic data, bioinformatic analyses and experimental results support DiiA as a promising immunoprotective antigen. Vaccination with this protein elicits IgG levels of different subclasses, reducing the nasopharyngeal colonization and conferring protection against systemic disease. Similar approaches may help to guard against infections caused by any pathogen, instead of exclusively concentrating the effort on traditional well-known proteins.

## Data Availability

Data is contained within the article or Supplementary Materials.

## Supplementary Materials

The following are available online at https://www.mdpi.com/2076-393X/9/3/187/s1, Figure S1: Verification of antiserum specificity by Western blot.
