# Supplementary material for: Immunization with SP_1992 (DiiA) Protein of Streptococcus pneumoniae Reduces Nasopharyngeal Colonization and Protects against Invasive Disease in Mice
Source: Vaccines (Basel). 2021 Feb 24;9(3):187. doi: 10.3390/vaccines9030187 (PMC7995960; doi:10.3390/vaccines9030187)
Supplement: Supplementary file 1 [file vaccines-09-00187-s001.pdf]

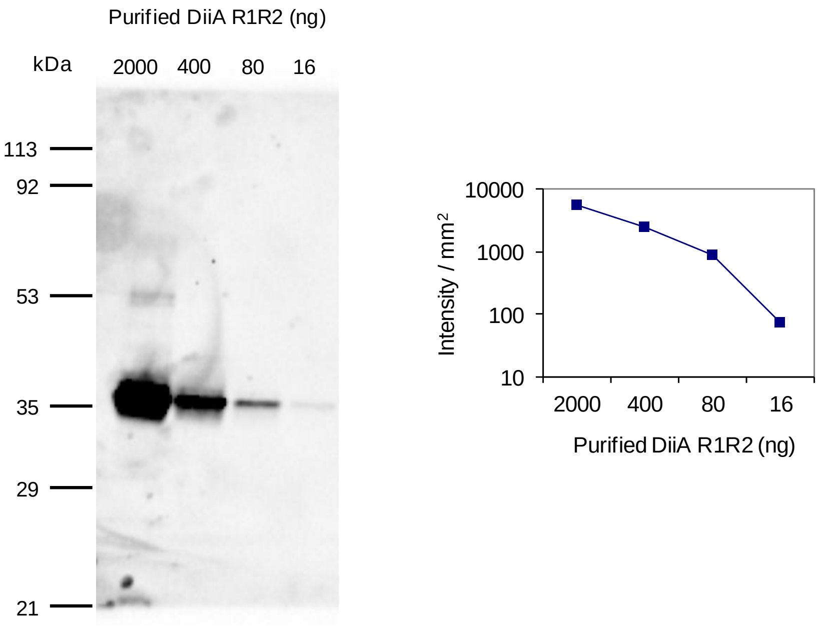

Supplemental Figure S1. Verification of antiserum specificity by Western blot. Western blot (left) and densitometry measurements of bands (right) are shown.
